# Heterogeneity of cerebral TDP-43 pathology in sporadic amyotrophic lateral sclerosis: Evidence for clinico-pathologic subtypes

**DOI:** 10.1186/s40478-016-0335-2

**Published:** 2016-06-23

**Authors:** Ryoko Takeuchi, Mari Tada, Atsushi Shiga, Yasuko Toyoshima, Takuya Konno, Tomoe Sato, Hiroaki Nozaki, Taisuke Kato, Masao Horie, Hiroshi Shimizu, Hirohide Takebayashi, Osamu Onodera, Masatoyo Nishizawa, Akiyoshi Kakita, Hitoshi Takahashi

**Affiliations:** Department of Pathology, Brain Research Institute, Niigata University, 1-757 Asahimachi-dori, Chuo-ku, Niigata 951-8585 Japan; Department of Neurology, Brain Research Institute, Niigata University, Chuo-ku, Niigata Japan; Department of Molecular Neuroscience, Brain Research Institute, Niigata University, Chuo-ku, Niigata Japan; Division of Neurobiology and Anatomy, Graduate School of Medicine and Dental Sciences, Niigata University, Chuo-ku, Niigata Japan

**Keywords:** Amyotrophic lateral sclerosis, Frontotemporal lobar degeneration, TDP-43, Cerebral cortex, Putamen, Globus pallidus, Dystrophic neurite, Dendrites

## Abstract

Frontotemporal lobar degeneration (FTLD) and amyotrophic lateral sclerosis (ALS) are types of major TDP-43 (43-kDa TAR DNA-binding protein) proteinopathy. Cortical TDP-43 pathology has been analyzed in detail in cases of FTLD-TDP, but is still unclear in cases of ALS. We attempted to clarify the cortical and subcortical TDP-43 pathology in Japanese cases of sporadic ALS (*n* = 96) using an antibody specific to phosphorylated TDP-43 (pTDP-43). The cases were divided into two groups: those without pTDP-43-positive neuronal cytoplasmic inclusions in the hippocampal dentate granule cells (Type 1, *n* = 63), and those with such inclusions (Type 2, *n* = 33). Furthermore, the Type 2 cases were divided into two subgroups based on semi-quantitative estimation of pTDP-43-positive dystrophic neurites (DNs) in the temporal neocortex: Type 2a (accompanied by no or few DNs, *n* = 22) and Type 2b (accompanied by abundant DNs, *n* = 11). Clinico-pathologic analysis revealed that cognitive impairment was a feature in patients with Type 2a and Type 2b, but not in those with Type 1, and that importantly, Type 2b is a distinct subtype characterized by a poor prognosis despite the less severe loss of lower motor neurons, the unusual subcortical dendrospinal pTDP-43 pathology, and more prominent glial involvement in cortical pTDP-43 pathology than other two groups. Considering the patient survival time and severity of motor neuron loss in each group, transition from Type 1 to Type 2, or from Type 2a to Type 2b during the disease course appeared unlikely. Therefore, each of these three groups was regarded as an independent subtype.

**Electronic supplementary material:**

The online version of this article (doi:10.1186/s40478-016-0335-2) contains supplementary material, which is available to authorized users.

## Introduction

Amyotrophic lateral sclerosis (ALS), an adult-onset, fatal neurodegenerative disorder, is the most common type of motor neuron disease (MND). Most cases (90-95 %) appear to occur randomly without a family history (sporadic ALS) [1, 2]. The principal feature is progressive muscular weakness due to degeneration of both the upper and lower motor neuron systems, and characteristic ubiquitin-positive neuronal cytoplasmic inclusions (NCIs) are present in the lower motor neurons [3–5]. It has also been well recognized that there are sporadic ALS cases accompanied by cognitive impairment (ALS or MND with dementia: ALS-D or MND-D), in which the presence of ubiquitin-positive NCIs and dystrophic neurites (DNs) in the extra-motor cortices, including the hippocampal dentate gyrus, is a significant feature [5–9]. Similar ubiquitin pathology has also been demonstrated in a subset of patients with frontotemporal dementia (FTD) with or without MND [10]. Accordingly, it has been suggested that ALS, ALS-D (MND-D) and FTD without MND represent a clinico-pathologic spectrum [6].

Since the identification of a nuclear protein, 43-kDa TAR DNA-binding protein (TDP-43, also known as TARDBP), as the major component of the ubiquitinated inclusions in frontotemporal lobar degeneration with ubiquitin-positive, tau-and α-synuclein-negative inclusions (FTLD-U) and ALS [11, 12], many studies of such cases employing TDP-43 immunohistochemistry have been performed [13–18]. This has led to the recognition that the clinico-pathologic spectrum encompassing ALS at one end and FTD at the other represents a new concept of TDP-43 proteinopathy [19, 20].

Cases of FTLD-U were originally divided into three types by two independent groups using their own classification systems based on the morphology and anatomical distribution of cortical ubiquitin neuronal lesions, including NCIs and DNs [21, 22]. After confirmation that the majority of FTLD-U cases in fact represent FTLD-TDP, these two classification systems were integrated into a ‘harmonized classification system’ that included four types (*Types A*, *B*, *C* and *D*) of FTLD-TDP pathology [23], the new *Type D* being associated with mutations of the *VCP* (valosin-containing protein) gene [24, 25]. In this new classification system, MND with FTD (so-called ALS-D) was regarded as a common phenotype of *Type B*, being characterized by moderate numbers of NCIs and very few DNs throughout all cortical layers. However, one of the above studies demonstrated that 7 of 10 cases that were sporadic and exhibited MND in addition to dementia had cortical ubiquitin pathology characterized by a mixture of numerous NCIs and frequent small DNs [22], corresponding to *Type A* of the new classification system mentioned above [23]; this finding suggested that the cortical ubiquitin pathology in ALS-D and/or FTLD-MND could be heterogeneous.

Using a phosphorylation-independent anti-TDP-43 antibody, we have previously demonstrated that immunopositive NCIs and glial cytoplasmic inclusions (GCIs) can occur in many brain regions in ALS, and that cases can be classified into two types – type 1 and type 2–based on the distribution pattern of NCIs in the CNS and hierarchical cluster analysis of the pattern [17]. Type 2 can be distinguished from type 1 by the presence of TDP-43-positive NCIs in the extra-motor neuron system, including the frontotemporal cortex, hippocampal formation, neostriatum and substantia nigra, and is significantly associated with dementia [17]. Since a monoclonal antibody specifically recognizing abnormally phosphorylated TDP-43 has become available, we have often noticed the presence of abundant threads, or dot-like or granular DNs in the temporal neocortex in cases of ALS, more strictly those with NCIs in the hippocampal dentate granule cells.

In the present study, we attempted to reevaluate the cortical and subcortical TDP-43 pathology in cases of sporadic ALS using the above monoclonal antibody, which never recognizes endogenous non-phosphorylated TDP-43 in nuclei, thus allowing unambiguous identification of pathologic structures. The results obtained eventually allowed us to classify the examined cases into three pathologic groups, whose clinical, pathologic and biochemical features were then analyzed.

## Materials and methods

The present study was conducted within the framework of a project, “Neuropathologic and Molecular-Genetic Investigation of CNS Degenerative Diseases”, approved by the Institutional Review Board of Niigata University. Informed consent was obtained from the patients’ families prior to genetic analyses.

### Subjects

We retrieved all cases of pathologically confirmed ALS from our institutional autopsy files covering the period between 1975 and 2013, reviewed the medical records and identified 128 cases of clinically sporadic ALS without any family histories of similar neurological disorders. All of the patients were of Japanese ancestry, and their clinical information was obtained retrospectively by reviewing their medical records.

Among these 128 cases, the tissue samples were of poor quality due to complications of infarction, etc. and/or sampling in 26 cases, pathologic features indicative of complications arising from other major neurodegenerative diseases affecting the cerebral cortex and basal ganglia were evident in 4 cases (Alzheimer’s disease = 2; progressive supranuclear palsy = 1; multiple system atrophy = 1), and no TDP-43-positive inclusions were detected in the CNS, including the lower motor neurons, in 2 cases. Accordingly, a total of 32 cases were excluded, leaving 96 cases (58 male, 38 female; mean age 67.4 years, standard deviation 9.8 years, range 36–87 years) for analysis. Seven cases were found to have only a few Lewy bodies, with α-synuclein-positive NCIs and DNs confined to the brainstem. These cases were considered to be incidental Parkinson’s disease and were included in the present study. All of the studied cases showed loss of upper and lower motor neurons as well as ubiquitin-positive skein-like inclusions in the remaining lower motor neurons, and Bunina bodies were evident in the remaining lower motor neurons in 91 of the 96 cases.

### Histology and immunohistochemistry

Multiple formalin-fixed, paraffin-embedded CNS tissue blocks for all cases were available for the present study. For the motor cortex, frontal cortex (including the prefrontal area), temporal cortex (including the hippocampus), basal ganglia, hypoglossal nucleus, and cervical and lumbar anterior horns, 4-μm-thick sections stained with hematoxylin-eosin (H-E) were used for semi-quantitative analysis employing a 4-point scale (0, absent; 1, mild; 2, moderate; 3, severe) of neuronal cell loss (Additional file 1: Figure S1). FTLD was diagnosed by the presence of atrophy and neuronal loss with gliosis in the frontotemporal cortices, regardless of severity. The study was carried out by two of the authors (R.T. and M.T.), and reviewed by two other investigators (Y.T. and H.T.) to ensure evaluation consistency.

Newly prepared 4-μm-thick sections were cut from the temporal cortex (including the hippocampus), frontal and motor cortices and basal ganglia for immunohistochemical studies. The sections were autoclaved at 120 °C in 10 mM citrate buffer, pH 6.0, for 10 min, and then immunostained with a mouse monoclonal antibody against phosphorylated TDP-43 (pTDP-43; phospho Ser409/410) (clone 11–9; Cosmo Bio Co., Ltd., Tokyo, Japan; 1:5000). Selected sections were also immunostained with a rabbit polyclonal phosphorylation-independent anti-TDP-43 antibody (10782-2-AP; Protein Tech Group Inc., Chicago, IL; 1:4000). Immunolabeling was detected using the peroxidase-polymer-based method using a Histofine Simple Stain MAX-PO kit (Nichirei Biosciences Inc, Tokyo, Japan) with diaminobenzidine (DAB) as the chromogen. To estimate the neuropathological staging of changes associated with Alzheimer’s disease, we performed Gallyas-Braak silver impregnation, and immunohistochemistry using mouse monoclonal antibodies against hyperphosphorylated tau protein (AT8; Innogenetics, Ghent, Belgium; 1:200) and β-amyloid (Dako, Glostrup, Denmark; 1:100). Then, we evaluated the Braak stages of neurofibrillary tangles and amyloid deposits [26, 27], and also estimated the level of Alzheimer’s disease-related neuropathologic change based on ‘ABC’ score [26–30].

### Classification procedure based on cortical pTDP-43 pathology

**Fig. 1:**
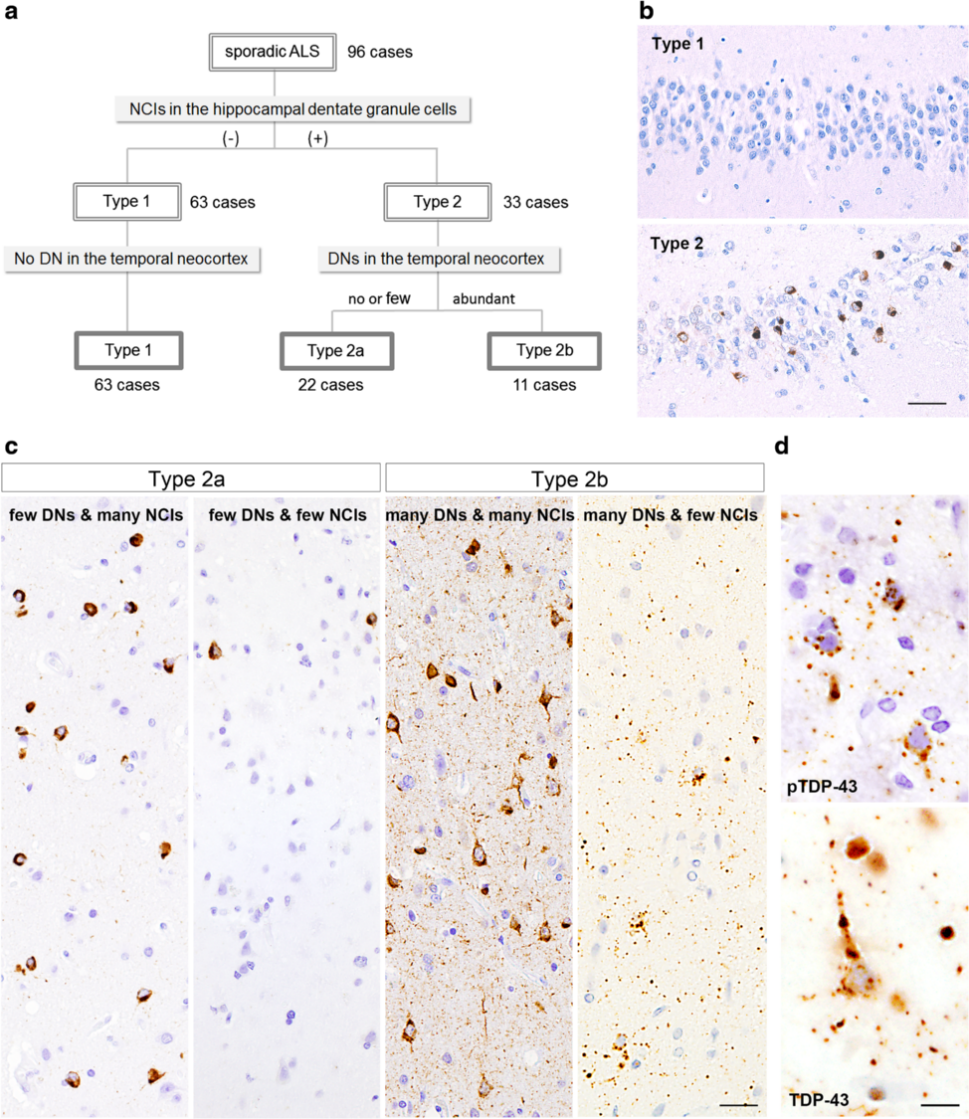
Procedure for classification of ALS-TDP pathology. (**a**) The procedure used for classification of the 96 cases of sporadic ALS and the results obtained. (**b**) Type 1 and Type 2 are defined by absence (upper) and presence (lower) of phosphorylated TDP-43 (pTDP-43) -positive NCIs in the hippocampal dentate granule cells, respectively. (**c**) Type 2a and Type 2b are defined by the presence of no or few, and abundant pTDP-43-positive DNs in the temporal neocortex, respectively. Representative TDP-43 pathology of Type 2a and Type 2b is shown. (**d**) NCIs and DNs demonstrated using anti-pTDP-43 antibody (upper) are also recognizable using phosphorylation-independent anti-TDP-43 antibody (lower). Scale bars: **b** = 40 μm; **c** = 25 μm; **d** = 10 μm

In our previous study of a series of 35 cases of sporadic ALS using a phosphorylation-independent antibody against TDP-43, we found that two pathologic phenotypes – type 1 and type 2 – were distinguishable as mentioned above, and that all cases showing TDP-43-positive NCIs in the hippocampal dentate granule cells were classifiable into type 2, whereas all cases except one showing no such NCIs were classifiable into type 1 [17]. Therefore, we first divided the 96 cases investigated in the present study into two groups: one without pTDP-43-positive NCIs in the dentate granule cells (Type 1, *n* = 63) and the other with such inclusions (Type 2, *n* = 33) (Fig. 1a, b). We then performed a semi-quantitative estimation of pTDP-43-positive DNs in the temporal neocortex of individual cases in each group. The DNs appeared almost exclusively as threads, and granular and dot-like structures. Cases in which many such pTDP-43-positive DNs were evident were classed as having abundant DNs, whereas cases in which such DNs were a much less prominent feature were classed as having no DNs, or few DNs, if any. Cases of Type 2 were divided into two subgroups: Type 2 accompanied by no or few DNs (Type 2a, *n* = 22) and Type 2 accompanied by abundant DNs (Type 2b, *n* = 11) (Fig. 1 a, c). No cases showed an “intermediate” density of DNs, or were unclassifiable to either subgroup. An important observation was that the density of pTDP-43-positive NCIs in the temporal cortex appeared to vary randomly, which meant that classification into specific groups on the basis of NCI density was not possible. In cases belonging to the other group, Type 1, no pTDP-43-positive DNs were evident in the temporal neocortex, and therefore further subdivision of this group was not possible at this stage. NCIs and DNs visualized using anti-pTDP-43 were also recognizable with phosphorylation-independent anti-TDP-43 (Fig. 1d).

### Double-labeling immunofluorescence

A double-labeling immunofluorescence study was performed to assess the anatomical localization of pTDP-43 deposits forming granular and dot-like DNs. Sections of the temporal lobe and basal ganglia, including the neostriatum and globus pallidus from three representative cases of Type 2b were examined using rabbit polyclonal anti-pTDP-43 (phospho Ser409/410) (Cosmo Bio Co., Ltd.; 1:2000) and mouse monoclonal anti-neurofilament H (non-phosphorylated) (SMI-32; Calbiochem, San Diego, CA; 1:500), as well as rabbit polyclonal anti-pTDP-43 (phospho Ser409/410) and mouse monoclonal anti-synaptophysin (Leica Biosystems; Newcastle-upon-Tyne, UK; 1:50). The second antibodies used were Alexa Fluor 488 goat anti-rabbit IgG and Alexa Fluor 555 goat anti-mouse IgG (Molecular Probes, Eugene, OR; 1:1000). The sections were treated with an Autofluorescence Eliminator Reagent (Millipore, Billerica, MA), mounted under glass coverslips using VectaShield mounting medium with 4,6-diamidino-2-phenylindole (DAPI) nuclear stain (Vector Laboratories, Burlingame, CA), and analyzed using a Carl Zeiss confocal laser scanning microscope (LSM700).

### Double labeling with in situ hybridization and immunohistochemistry

The numbers of neurons and glial cells possessing cytoplasmic pTDP-43-positive inclusions (NCIs and GCIs) were assessed in the motor cortex using a double-labeling method with in situ hybridization (ISH) and immunohistochemistry, and compared between the three groups. For this study, we selected representative cases of Type 1 (*n* = 22), Type 2a (*n* = 12) and Type 2b (*n* = 7) among cases logged after 1990. In the Type 1 cases, a significant number of pTDP-43-positive NCIs and GCIs were seen in the motor cortex. Therefore, from the Type 2a and Type 2b cases, we selected cases in which a larger number of pTDP-43-positive NCIs and GCIs were evident in the motor cortex than in the temporal or frontal cortex.

ISH was performed in these three groups using newly prepared paraffin-embedded 10-μm-thick sections from the motor cortex, as described previously [31], with minor modifications (Supplementary Methods). A probe for human *neurofilament 3* (*hNF*: 150-kDa medium) (GenBank accession number: BC002421) was used. As a result, the sections from 11 cases were found to be inadequate for ISH, leaving 30 cases (Type 1, *n* = 16; Type 2a, *n* = 8; Type 2b, *n* = 6) logged between 1990 and 2012 for the subsequent immunohistochemical study.

The hybridized sections were immunostained using mouse monoclonal anti-pTDP-43 (clone 11–9; 1:5000), and then counterstained with Nuclear Fast Red solution (Sigma Aldrich, St. Louis, MO). In each case, 10 sequential images of the motor cortex were taken through a × 20 objective lens using a single ISH-labeled and immunostained section. The total area taken from a section from each case was 1.5 mm^2^, covering the cortical layers II-VI. The numbers of *hNF*-positive cells with nuclei, *hNF*- and pTDP-43-positive cells with nuclei, and *hNF*-negative and pTDP-43-positive cells with nuclei were counted manually.

### Statistical analysis

To compare clinical and pathologic features between the three groups, we used Kruskal-Wallis test with post-hoc Steel-Dwass test for non-parametric analysis of independent samples, multiple regression analysis to determine whether independent variables can predict the value of the dependent variable, Kaplan-Meier plots and a log-rank test to compare survival distributions, and Fisher’s exact test with Bonferroni-corrected multiple comparisons or Ryan’s multiple comparison test for comparison of categorical data. These statistical analyses were performed using GraphPad Prism version 5.0 (GraphPad Software, San Diego, CA), SPSS Statistics version 12.0 (IBM, Armonk, NY) and R version 3.1.2 (http://www.r-project.org/). Differences were considered statistically significant at *P* <0.05.

### Biochemical analysis of pTDP-43

Fractionation of frozen brain tissues and TDP-43 immunoblotting were performed on selected cases. Protein lysates were generated from the motor cortex of Type 1 (*n* = 4) cases and from the temporal cortex of Type 2a (*n* = 4) and Type 2b (*n* = 4) cases, as well as from the frontal cortex of FTLD-TDP *Type A*, *B* and *C* (two each), as described previously [32–34], with minor modifications (Additional file 2). In order to distinguish the 20-25-kDa band pattern clearly, we used a large polyacrylamide gel (184 × 185 mm) and electrophoresed the samples at 200 V for 16 h at 4 °C. The separated samples were analyzed by immunoblotting with a mouse monoclonal antibody against pTDP-43 (clone 11–9; 1:2000) [35].

### Analysis of the TARDBP and C9ORF72 genes

The presence or absence of *TARDBP* and *C9ORF72* gene mutations was analyzed in cases for which frozen tissue samples were available. High-molecular-weight genomic DNA was extracted from 83 cases (Type 1, *n* = 54; Type 2a, *n* = 19; Type 2b, *n* = 10). We amplified all the exons of the *TARDBP* (NM_007375.3) gene using a series of primers, followed by sequence reaction [36]. For screening of GGGGCC repeat expansion in *C9ORF72* (NM_018325.2), repeat-primed PCR was performed on an ABI 3130xl genetic analyzer (Applied Biosystems, Foster City, CA) using Peak Scanner software v1.0 (Applied Biosystems), as described previously [37, 38].

## Results

### Clinical features

The demographics and clinical features of the studied patients are summarized in Table 1. There was no evident difference in gender or cause of death between patients with Type 1, and those with Type 2a and Type 2b. However, statistical comparison among the three groups revealed that the age at onset was higher for Type 2b than for Type 1 (*P* = 0.024), and that the survival time from disease onset was shorter for Type 2b than for Type 1 (*P* <0.0001) and Type 2a (*P* = 0.003). Multiple regression analysis using age at onset and ALS subtype as independent covariates and the survival time from disease onset as a dependent variable, a significant correlation was found between ALS subtype and the survival time from disease onset (*P* = 0.039), whereas the correlation for age at onset was not statistically significant (*P* = 0.106). Comparison of initial symptoms between the three groups showed that limb weakness was more frequent whereas bulbar and other symptoms were significantly less frequent in Type 1 than that in Type 2a (*P* <0.0001) and Type 2b (*P* <0.01). Cognitive impairment was present in 15 (16 %) of the 96 patients, the incidence being similar to that (10-15 %) of overt cognitive impairment meeting the criteria for frontotemporal dementia reported previously in patients with ALS [39]. The rate of occurrence of cognitive impairment was lower in Type 1 than in Type 2a (*P* <0.001) and Type 2b (*P* <0.00001). There were no evident differences in the age at death between cases with (*n* = 15) and those without (*n* = 81) cognitive impairment (Mean ± SD: 67.6 ± 7.5 vs 67.4 ± 10.2 years, *P* = 0.959).

**Table 1:** Demographics and clinical features in three types of sporadic ALS

	All	Type 1	Type 2a	Type 2b	*P*-value
	(*n* = 96)	(*n* = 63)	(*n* = 22)	(*n* = 11)	
Sex (male: female)	58:38	38:25	13:9	7:4	0.968
Age at onset^a^ (years)	64.5 (32–86)	62.0 (32–86)	65.5 (50–79)	72.0* (59–82)	0.023
Survival time^a^ (months)	22 (6–204)	33 (7–204)	20 (6–64)	12** (9–24)	<0.0001
Initial symptoms					<0.0001
limb	61 (64 %)	50 (79 %)***	7 (32 %)	4 (36 %)	
bulbar	32 (33 %)	13 (21 %)	13 (59 %)	6 (55 %)	
others^b^	3 (3 %)	0	2 (9 %)	1 (9 %)	
Cognitive impairment	15 (16 %)	1 (2 %)****	7 (32 %)	7 (64 %)	<0.0001
Cause of death					0.793
respiratory failure	67 (70 %)	45 (71 %)	14 (64 %)	8 (73 %)	
others^c^	29 (30 %)	18 (29 %)	8 (36 %)	3 (27 %)	

*ALS* amyotrophic lateral sclerosis

^a^Data are expressed as median (range)

^b^Three patients developed cognitive impairment or character change before the appearance of motor symptoms. Motor symptoms became evident seven months after disease onset in one patient (Type 2a), and within a month in the other two patients (Type 2a and Type 2b, respectively)

^c^Infections, gastrointestinal bleeding or sudden death

*P = 0.024 vs. Type 1 (Kruskal-Wallis test with post-hoc Steel-Dwass test), **P <0.0001 vs. Type 1 and *P* = 0.003 vs. Type 2a (Kaplan-Meier method and log-rank test), ***P <0.0001 vs. Type 2a and *P* <0.01 vs. Type 2b (Fisher’s exact test with Bonferroni-corrected multiple comparison test), ****P <0.001 vs. Type 2a and *P* <0.00001 vs. Type 2b (Fisher’s exact test with Ryan’s multiple comparison test)

### Pathologic features

The pathologic features of the studied cases are summarized in Table 2. In the motor cortex, there were no evident differences in the severity of neuronal loss among the three groups: Type 1, and Type 2a and Type 2b. However, in the spinal anterior horns (cervical and lumbar) and hypoglossal nucleus, loss of motor neurons was less severe in Type 2b than in Type 1 (*P* <0.001 and *P* = 0.001, respectively) or in Type 2a (*P* = 0.018 and *P* = 0.022, respectively). No neuronal loss in the basal ganglia was evident in any of the cases. None of the present cases showed hippocampal sclerosis characterized by neuronal loss and gliosis in the hippocampal formation, which is found often in FTLD-TDP but rarely in ALS [40, 41]. The Additional file 3: Table S1 shows the neuropathological stages of Alzheimer’s disease-associated changes in all of the 96 cases. Eighty-six, 10, and 0 cases corresponded to Braak neurofibrillary stage 0-II, stage III, and stage IV-VI, respectively. In accordance with the ‘ABC’ score [29], 93 and 3 cases corresponded to ‘low’ and ‘intermediate’ levels of Alzheimer’s disease neuropathologic change, respectively. The three patients exhibiting ‘intermediate’ change showed no cognitive impairment.

**Table 2:** Pathologic features and phosphorylated TDP-43 pathology in three types of sporadic ALS

	Type 1	Type 2a	Type 2b	*P*-value
	(*n* = 63)	(*n* = 22)	(*n* = 11)	
Neuronal loss^a^				
Motor cortex	2.1 ± 0.7	2.1 ± 0.7	2.5 ± 0.7	0.073
Anterior horns (cervical and lumbar)	2.2 ± 0.5	2.1 ± 0.6	1.5 ± 0.4*	0.002
Hypoglossal nucleus	2.3 ± 0.7	2.1 ± 0.7	1.4 ± 0.5**	<0.001
Frontotemporal degeneration (cases)	4 (6 %)***	8 (36 %)	7 (64 %)	<0.0001
pTDP-43 pathology (cases)				
NCIs: temporal-predominant type	NA	13 (59 %)	5 (45 %)	NA
DNs: many in neostriatum and globus pallidus	1 (2 %)	2 (9 %)	9 (82 %)****	<0.0001

*ALS* amyotrophic lateral sclerosis, *pTDP*-43 phosphorylated TDP-43, *NCI* neuronal cytoplasmic inclusion, *DN* dystrophic neurite, *NA* non-applicable

^a^0 = absent, 1 = mild, 2 = moderate, 3 = severe. Data are expressed as mean ± standard deviation

*P <0.001 vs. Type 1 and *P* = 0.018 vs. Type 2a, **P = 0.001 vs. Type 1 and *P* = 0.022 vs. Type 2a (Kruskal-Wallis test with post-hoc Steel-Dwass test), ***P <0.001 vs. Type 2a and *P* = 0.00001 vs. Type 2b (Fisher’s exact test with Ryan’s multiple comparison test), ****P <0.00001 vs. Type 1 and *P* <0.0001 vs. Type 2a (Fisher’s exact test with Ryan’s multiple comparison test)

In each group, a proportion of cases showed FTLD manifested by frontotemporal atrophy and frontotemporal cortical neuronal loss with gliosis; the rate of occurrence of FTLD was only 6 % in Type 1, being much lower than in Type 2a (36 %, *P* <0.001) or in Type 2b (64 %, *P* = 0.00001). In around half of the cases in both the Type 2a and Type 2b groups, pTDP-43-positive NCIs showed a temporal cortex-predominant distribution pattern, in comparison with the motor cortex. No pTDP-43-positive neuronal intranuclear inclusions were evident in any of the cases examined.

**Fig. 2:**
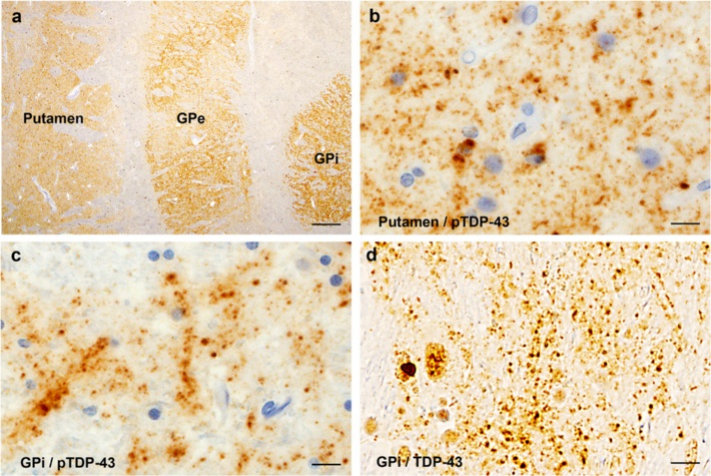
Dendrospinal pTDP-43 pathology in the neostriatum and globus pallidus. (**a**) Low-magnification view of the basal ganglia from a case of Type 2b delineates the putamen and globus pallidus with phosphorylated TDP-43 (pTDP-43) immunoreactivity. (**b**, **c**) High-magnification views of the putamen (**b**) and globus pallidus (**c**) show numerous pTDP-43-positive granular and dot-like structures. (**d**) Similar granular and dot-like structures in the globus pallidus are also visualized using phosphorylation-independent anti-TDP-43. Scale bars: **a** = 500 μm; **b**-**d** = 10 μm. GPe = external segment of globus pallidus; GPi = internal segment of globus pallidus

In the subcortical region, pTDP-43-positive granular and dot-like DNs were a feature of the neostriatum and globus pallidus in most cases of Type 2b (82 %) (Table 2 and Fig. 2a-c). The rate of occurrence of such pTDP-43 pathology was significantly lower, being only 2 % and 9 %, in Type 1 (*P* <0.00001) and Type 2a (*P* <0.0001), respectively, than in Type 2b. These inclusions were also detectable with the phosphorylation-independent anti-TDP-43 antibody (Fig. 2d).

### The anatomical localization of pTDP-43 deposits forming granular and dot-like DNs

**Fig. 3:**
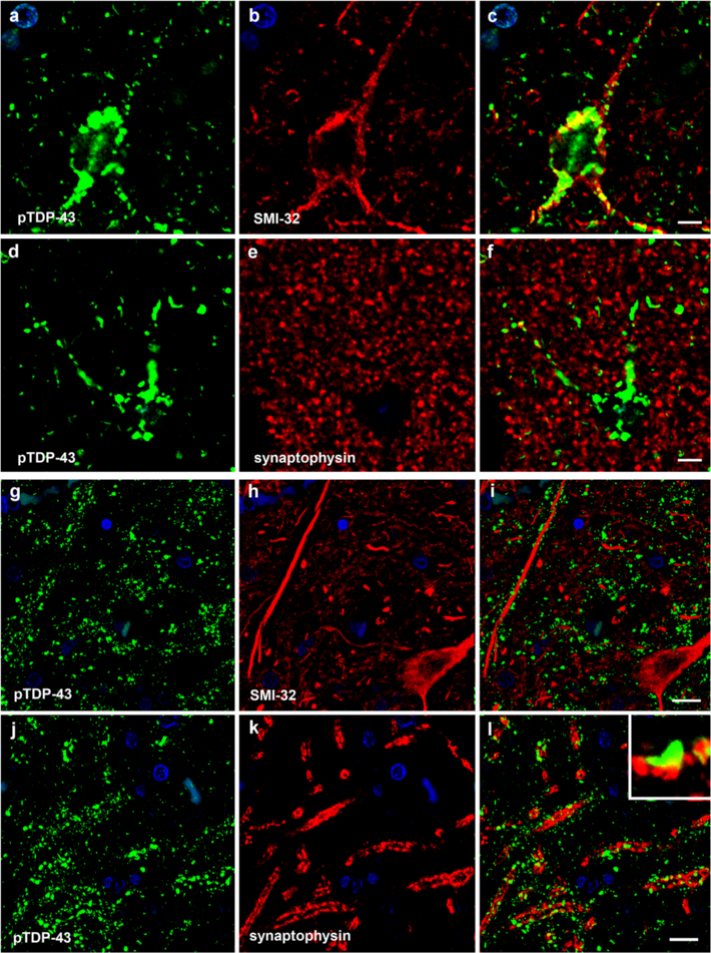
The anatomical localization of pTDP-43 deposits forming granular and dot-like DNs. (**a**-**l**) Double-labeling immunofluorescence in the temporal cortex (**a**-**f**) and globus pallidus (**g**-**l**) from two different cases of Type 2b. Phosphorylated TDP-43 (pTDP-43)-positive granular and dot-like structures are observed along SMI-32-positive dendrites (**a**, **g**: green, pTDP-43; **b**, **h**: red, SMI-32; **c**, **i**: merged). No co-localization of pTDP-43 and synaptophysin is evident (**d**, **j**: green, pTDP-43; **e**, **k**: red: synaptophysin; **f**, **l**: merged). Close association with pTDP-43-positive structures and synaptophysin-positive structures is observed in the globus pallidus (**j**-**l**). There is evidence of contact between pTDP-43- and synaptophysin-positive structures, suggesting that pTDP-43 is localized in the dendritic spines (**l**, insert). Scale bars: **a**-**f** = 5 μm; **g**-**l** = 10 μm

SMI-32 and synaptophysin are known to be dendritic and presynaptic markers, respectively. In the temporal cortex and globus pallidus, pTDP-43-positive granular and dot-like structures were observed along the SMI-32-positive dendrites (Fig. 3a-c and g-i, respectively), and it was evident that co-localization of pTDP-43 and synaptophysin was not a feature (Fig. 3d-f and j-l, respectively). Moreover, in the globus pallidus, it was clear that pTDP-43-positive granular and dot-like structures were located closely beside synaptophysin-positive structures (Fig. 3j-l). In the putamen, we observed a localization pattern of pTDP-43 similar to that in the temporal cortex and globus pallidus (data not shown). These results strongly suggested that deposition of the pTDP-43 occurred in the dendritic spines.

### pTDP-43 inclusion-bearing neurons and oligodendrocytes in the motor cortex

**Fig. 4:**
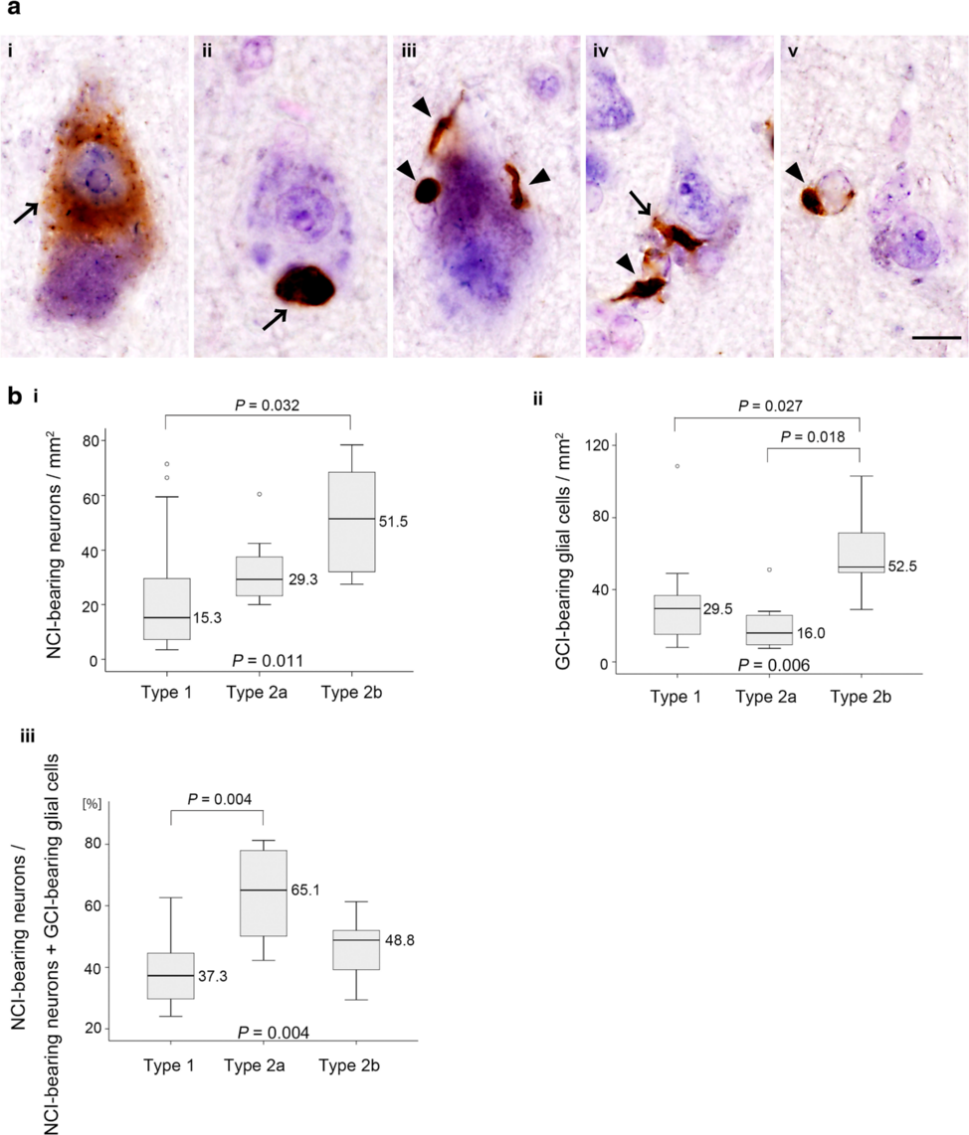
Neuronal and glial pTDP-43 pathology in the motor cortex. (**a**) Double labeling with *human neurofilament* in situ hybridization (blue) and phosphorylated TDP-43 (pTDP-43) immunohistochemistry (brown). High-magnification views of pTDP-43-positive NCIs (arrows) and GCIs (arrowheads) of various shapes in the motor cortex (**i**-**v**). pTDP-43-positive GCIs occur exclusively in oligodendrocytes (**iii**-**v**), including those adjacent to neurons (satellite oligodendrocytes) (**iii**, **iv**). Scale bar = 10 μm for **i**-**v**. (**b**) Box-plots indicate the density of NCI-bearing neurons (**i**) as well as that of GCI-bearing glial cells (**ii**), and the ratio of NCI-bearing neurons to total NCI-bearing neurons and GCI-bearing glial cells (**iii**). Minimum and maximum numbers or percentages are depicted by short horizontal lines, the box signifies the upper and lower quartiles, and the median is represented by a long horizontal line within the box for each group. *P*-values shown at the bottom and top of each graph were calculated by Kruskal-Wallis test and post-hoc Steel-Dwass test, respectively

Next, to investigate the density of NCI-bearing neurons and GCI-bearing oligodendrocytes, we performed double labeling with in situ hybridization using a probe for *hNF* (mRNA) and immunohistochemistry using an antibody for pTDP-43 (protein). Brain cells positive for *hNF* were recognized as neurons (Fig. 4a i-v), and pTDP-43 inclusion-bearing brain cells negative for *hNF* were considered to be glial cells, or more strictly oligodendrocytes (Fig. 4a iii-v). There was no evidence of any perivascular distribution of pTDP-43-positive inclusions suggestive of astrocytic involvement (Fig. 1c); we did not take astrocytes into consideration.

The density of NCI-bearing neurons was higher in Type 2b than in Type 1 (*P* = 0.032) (Fig. 4b i), although there was no difference in the total number of neurons between the two; the median neuron count per 1 mm^2^ was 553.8 (interquartile range: 503.7-577.8) for Type 1, 573.7 (516.7-619.7) for Type 2a, and 575.4 (533.3-650.3) for Type 2b, *P* = 0.316. The density of GCI-bearing glial cells was higher in Type 2b than that in Type 1 (*P* = 0.027) and Type 2a (*P* = 0.018) (Fig. 4b ii). The ratio of NCI-bearing neurons to total NCI-bearing neurons and GCI-bearing glial cells was higher in Type 2a than in Type 1 (*P* = 0.004) (Fig. 4b iii), although there was no difference in the density of NCI-bearing neurons between the two (*P* = 0.114) (Fig. 4b i).

### Biochemical and genetic features

**Fig. 5:**
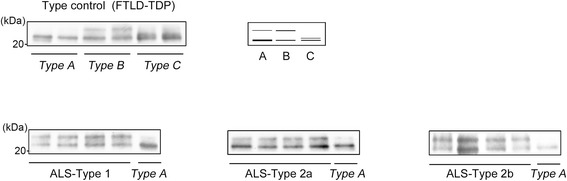
pTDP-43 immunoblot analysis. Immunoblot analysis of sarkosyl-insoluble fractions from the affected cortex of FTLD-TDP *Type A*, *B*, and *C* cases (two each; upper panels), and Type 1, Type 2a and Type 2b cases (four each; lower panels) immunostained with anti- phosphorylated TDP-43 antibody (pTDP-43, phospho Ser409/410). In all cases from the three ALS groups, both the upper (24-kDa) and lower (23-kDa) bands of the C-terminal fragments of pTDP-43 are clearly detected, and the pattern of these fragments is similar to those shown in FTLD-TDP *Type B*, and not in FTLD-TDP *Type A* or *Type C*

The data obtained by immunoblot analysis of sarkosyl-insoluble fractions are shown in Fig. 5. The anti-phosphorylated TDP-43 antibody detected a ~45-kDa band and a ~26-kDa fragment in all cases of ALS and FTLD-TDP examined. Focusing on the pattern of the ~26-kDa bands, in all cases of sporadic ALS examined, both the upper (24-kDa) and lower (23-kDa) bands of the C-terminal fragments of pTDP-43 were clearly detected, and found to be compatible with those obtained in two cases of FTLD-TDP *Type B*, and different from those in FTLD-TDP *Type A*, showing no evident differences between the three ALS groups.

With regard to the sequencing of the TDP-43 (*TARDBP*) gene in the 83 cases examined, there were no mutations in the coding regions except for one case with a known synonymous mutation in exon 6 [36]. No *C9ORF72* repeat expansion was observed.

## Discussion

In the present study, a series of 96 cases of sporadic ALS were classified into three groups on the basis of the cortical pTDP-43 pathology (Fig. 1a). Based on the absence or presence of pTDP-43-positive NCIs in the hippocampal dentate granule cells, we divided all cases into two groups, Type 1 and Type 2 (Fig. 1a, b). Among the cases belonging to Type 2, we noted that some were characterized by the presence of DNs in the affected temporal neocortex in addition to NCIs (Fig. 1a, c). Accordingly, we further divided these Type 2 cases into two subgroups, i.e. Type 2a and Type 2b, based on the presence or absence of the prominent appearance of pTDP-43-positive DNs, irrespective of the amount of NCI-bearing neurons. Interestingly, statistical analysis revealed a number of significant differences of clinico-pathologic features among these three groups, which appeared to support the validity of this classification (Tables 1, 2).

Comparison of the ubiquitin- or TDP-43-related cortical pathology of FTLD described by many investigators suggested that Type 2a apparently corresponded to *Type B* in the harmonized classification system for FTLD-TDP pathology reported by Mackenzie et al. (2011) [16, 21, 22, 40, 42], whereas Type 2b showed a very unusual cortical TDP-43 pathology characterized by many DNs in the form of threads, and granular and dot-like structures (Fig. 1c). This pathologic picture appeared to be somewhat similar to, but by no means consistent with that of *Type A* in the classification system mentioned above (the majority of DNs observed in cases classified as Type 2b appeared to be smaller structures). Furthermore, immunoblot analysis of sarkosyl-insoluble pTDP-43 revealed that the band patterns of Type 2b and Type 1 were indistinguishable and differed from those of FTLD-TDP *Type A* (Fig. 5). By contrast, *Type A* pathology and a FTLD-TDP *Type A* band pattern have been described in the motor cortex in two cases of primary lateral sclerosis (PLS) [33]. Therefore, we conclude that, to our knowledge, the overall picture of Type 2b is distinct from those observed in patients with FTLD-U or FTLD-TDP.

From a clinico-pathologic viewpoint, cognitive impairment was much less frequent in patients with Type 1 as we suggested previously [17]; in other words, its presence in patients with sporadic ALS strongly suggests Type 2a or Type 2b. Moreover, Type 2b, although accounting for only a small proportion of the disease spectrum, could be regarded as a distinct subset showing a relatively old age at onset and a shorter survival time than the other two groups. Patients in this group frequently developed bulbar symptoms initially (Table 1). From a pathologic viewpoint, less severe loss of lower motor neurons and the presence of numerous pTDP-43-positive granular and dot-like DNs in the putamen and globus pallidus were notable features in Type 2b (Table 2).

In the present study, clinical information was limited to that obtained retrospectively from the medical records of the patients for whom details of some clinical symptoms were unavailable. This was one of the limitations of the present study. It has been shown that classification of patients with ALS based on clinical assessment provides information for better understanding the wide spectrum of this motor and cognitive disorder [43]. Moreover, neuropathologic examination of a large-scale cohort of clinically assessed patients would be expected to reveal pathognomonic features responsible for the various symptoms.

The results of double-labeling immunofluorescence using anti-pTDP-43 antibody and dendritic (SMI-32) or presynaptic (synaptophysin) markers in the temporal cortex and basal ganglia of patients with Type 2b suggested that deposition of pTDP-43 might occur in neuronal dendritic spines (Fig. 3). TDP-43 is normally localized primarily to the nucleus, where it regulates transcription and alternative splicing [12, 32]. In addition, recently, the roles of TDP-43 in dendrites and spines have been successively investigated [44, 45]. In neurons, TDP-43 is expressed abundantly in dendrites mainly in the form of RNA granules, and translocation of the TDP-43-containing granules into dendritic spines in response to neuronal activities has been demonstrated in cultured neurons. Accordingly it has been proposed that TDP-43 is a neuronal activity-responsive factor functioning in the regulation of neuronal plasticity [45]. More recently, it has been reported that disease-linked mutations in TDP-43 alter the dynamics of neuronal RNA granules during dendritic arborization, and reduce or slow the response of TDP-43 to changes in neuronal activity [44]. Considering these findings, it could be speculated that accumulation of the pathologic protein, pTDP-43, in neuronal dendritic spines of the cerebral cortex, as well as in the neostriatum and globus pallidus, leads to a decline in synaptic plasticity, and is at least partly responsible for the poor prognosis of patients with Type 2b, mainly through respiratory failure, despite the fact that loss of lower motor neurons is milder than that seen in patients with Type 1 and Type 2a.

Comparison of the TDP-43 cellular pathology in the motor cortex among the three disease groups showed that both the density of NCI-bearing neurons and GCI-bearing glial cells were higher in Type 2b than in Type 1. In addition, the ratio of NCI-bearing neurons to all inclusion-bearing cells was higher in Type 2a than in Type 1. Propagation of pathologic TDP-43 via axonal connections has been proposed on the basis of findings from autopsied ALS brains [46], and it has also been demonstrated that oligodendroglial involvement by pathologic TDP-43 precedes neuronal involvement in the spinal cord in ALS [47]. However, the mechanisms responsible for the spread of TDP-43 pathology into multiple systems of the CNS remain uncertain. Our present findings indicate that the mechanism of cellular involvement in propagation differs between Type 2b and Type 2a, oligodendrocytes also being substantially involved, in addition to neurons, in the former.

We consider that the three groups of sporadic ALS described here are originally independent of each other. Based on the TDP-43 pathologic staging scheme proposed by Brettschneider et al. [46], all cases of Type 2 in the present study corresponded to Stage 4, and the TDP-43 pathology appeared to progress from Stage 1 to 4 during the disease course. However, in sporadic ALS, we have previously demonstrated that long-term survival facilitated by artificial respiratory support has no apparent influence on the distribution pattern of neuronal inclusions in the two types (type 1 and type 2) of TDP-43 [17], and that 5 of 6 patients who survived for 10–20 years without respiratory support showed type 1 TDP-43 pathology [48]. Moreover, the present study revealed that survival time was shorter and that loss of lower motor neurons was less severe in patients with Type 2b than in those suffering from Type 1 and Type 2a. Taken together, we concluded that transition from Type 1 to Type 2, or from Type 2a to Type 2b, depending on disease duration or progression, would be unlikely to occur.

None of the patients in the present study had mutations in *C9ORF72*. Hexanucleotide (GGGGCC) repeat expansion in a non-coding region of *C9ORF72* is the major genetic cause of FTD and ALS (c9FTD/ALS) in the Caucasian population [49], but extremely rare in Japan [38, 50, 51]. When considering the rarity of this genetic disease in Japan, it is interesting to note that the reported national incidence rate of ALS in 2009 was 2.3 per 100,000 population [52], being much lower than the rates reported for Caucasian populations in Europe and North America [53].

## Conclusion

In conclusion, we have demonstrated heterogeneity of cortical pTDP-43 pathology in sporadic ALS, allowing the disease to be classified into three groups: Type 1, and Type 2a and Type 2b. Several significant clinical and pathologic correlations among these groups were evident. It was re-confirmed that dementia is not a feature of Type 1. Furthermore, the distinct subtype, Type 2b, accompanied by dendrospinal accumulation of pTDP-43, was shown to be characterized by a poor prognosis despite the less severe loss of lower motor neurons, the unusual subcortical dendritic pTDP-43 pathology, and more prominent glial involvement in cortical pTDP-43 pathology than other two groups. Further studies will be needed in order to establish the character of this subtype, Type 2b. Recognition of the heterogeneity of the anatomical and cellular localization of pTDP-43 will be important for clarifying the pathomechanisms involved in pTDP-43 aggregation and propagation in sporadic ALS, and eventually for developing future strategies of treatment and prevention.

## Abbreviations

ALS, amyotrophic lateral sclerosis; DN, dystrophic neurite; FTLD, frontotemporal lobar degeneration; GCI, glial cytoplasmic inclusion; MND, motor neuron disease; NCI, neuronal cytoplasmic inclusion; pTDP-43, phosphorylated 43-kDa TAR DNA-binding protein

## Additional files

Additional file 1: Figure S1. The degree of neuronal cell loss determined using a semi-quantitative approach. (TIF 45337 kb) Additional file 2: Contains supplementary methods regarding ISH and biochemical studies, and legends to Supplementary Figure 1. (DOC 36 kb) Additional file 3: Table S1. Supplementary results on the neuropathological stages of Alzheimer’s disease-associated changes. (DOC 38 kb)
