# Supplementary figures and images for: Heterogeneity of cerebral TDP-43 pathology in sporadic amyotrophic lateral sclerosis: Evidence for clinico-pathologic subtypes
Source: Acta Neuropathol Commun. 2016 Jun 23;4:61. doi: 10.1186/s40478-016-0335-2 (PMC4918136; doi:10.1186/s40478-016-0335-2)

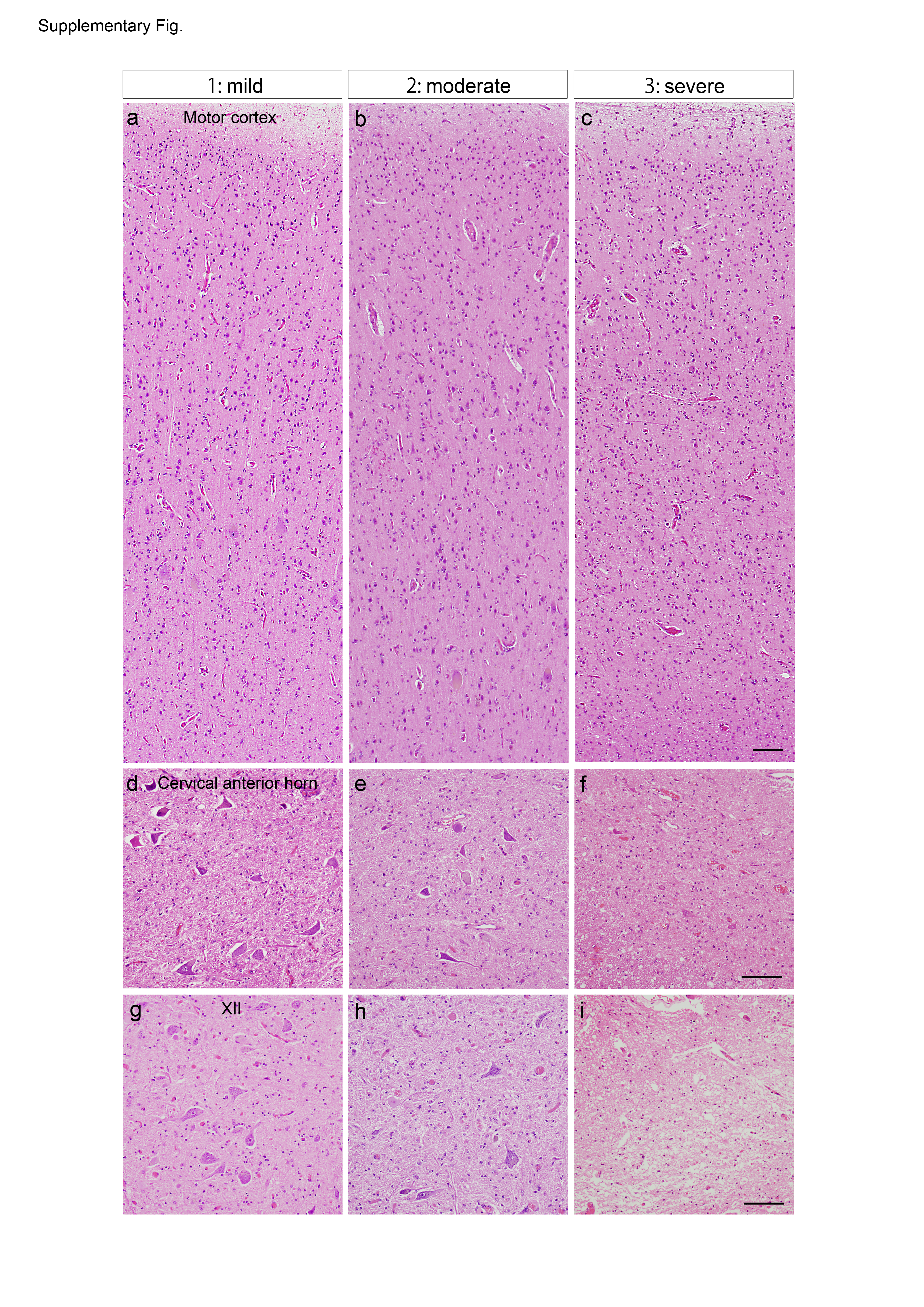

Supplement: Additional file 1: Figure S1. — The degree of neuronal cell loss determined using a semi-quantitative approach. (TIF 45337 kb) [file 40478_2016_335_MOESM1_ESM.tif]
