# Supplementary material for: Heterogeneity of cerebral TDP-43 pathology in sporadic amyotrophic lateral sclerosis: Evidence for clinico-pathologic subtypes
Source: Acta Neuropathol Commun. 2016 Jun 23;4:61. doi: 10.1186/s40478-016-0335-2 (PMC4918136; doi:10.1186/s40478-016-0335-2)
Supplement: Additional file 2: — Contains supplementary methods regarding ISH and biochemical studies, and legends to Supplementary Figure 1. (DOC 36 kb) [file 40478_2016_335_MOESM2_ESM.doc]

**Additional file 2**

**Supplementary Methods**

***In situ hybridization***

Deparaffinized sections were rehydrated through xylene and ethanol into phosphate-buffered saline (PBS) and treated with 5 µg/ml proteinase K in Tris-based buffer [50 mM Tris-HCl (pH 7.6), 5 mM EDTA] for 90 min, and then rinsed in 0.01 M PBS. After fixation in 4% paraformaldehyde in 0.01 M PBS and acetylation in 0.1 M triethanolamine (pH 8.0) containing 0.25% acetic anhydride for 10 min, the sections were prehybridized at 65 °C for 3 h with hybridization solution: 50% formamide, 5×saline sodium citrate (SSC) (1×SSC is 0.15 M NaCl, 0.015 M sodium citrate in diethylpyrocarbonate-treated water), 0.2 mg/ml yeast tRNA, 0.1 mg/ml heparin, 1×Denhardt’s solution, 0.1% Tween 20, 0.1% CHAPS, and 5 mM EDTA. The sections were then incubated with hybridization solution containing diluted digoxigenin-labeled RNA probe at 65 °C overnight. The hybridized sections were washed three times with 1×SSC and 50% formamide at 65 °C for 15 min (wash I) and 30 min (wash II), and with 0.1×SSC at 65 °C for 30 min (wash III). The sections were washed twice with maleic acid buffer [0.1 M maleic acid (pH 7.5), 0.15 M NaCl and 0.1% Tween 20] for 30 min at room temperature and incubated with alkaline phosphatase-conjugated sheep anti-digoxigenin antibody (Roche Diagnostics; Manheim, Germany; 1:2000) overnight at 4 °C. They were then washed in three changes of maleic acid buffer for 30 min each, and incubated with the color development solution [50 µg/ml 4-nitro blue tetrazolium chloride and 175 µg/ml 5-bromo-4-chloro-3-indolyl-phosphate (Roche Diagnostics)] in alkaline phosphatase buffer [0.1 M Tris-HCl (pH 9.5), 0.05 M MgCl_2_, 0.1 M NaCl and 0.1% Tween 20] for 5–24 h in the dark.

***Biochemical analysis of pTDP-43***

Frozen tissues were homogenized in 18 volumes of buffer H (10 mM Tris-HCl, 1 mM EGTA, 0.8 M NaCl and 10% sucrose) with 5 mM sodium orthovanadate and 1 mM sodium fluoride followed by the addition of 10% sarkosyl buffer for a final concentration of 2%. These homogenates were incubated for 30 min at 37 °C followed by sonication and centrifuged at 10000 rpm for 10 min at 25 °C. The supernatants were centrifuged at 50000 rpm for 20 min at 25 °C. The sarkosyl-insoluble pellets were sonicated in 30 mM Tris-HCl (pH 7.5) and boiled with laemmi sample buffer for 4 min. After centrifugation at 14000 rpm for 3 min at room temperature, the supernatants were collected as the sarkosyl-insoluble fractions. These samples were separated by sodium dodecyl sulfate-polyacrylamide gel electrophoresis and analyzed by immunoblotting with the mouse monoclonal anti-phosphorylated TDP-43 antibody (pTDP-43, clone 11-9; 1:2000).

**Supplementary figure legend**

**Fig. S1 The degree of neuronal cell loss determined using a semi-quantitative approach**

Examples of 1 (mild), 2 (moderate) and 3 (severe) of neuronal cell loss in the motor cortex (**a-c**), cervical anterior horn (**d-f**) and hypoglossal nucleus (**g-i**). Scale bars = 100 μm.
