# Supplementary material for: Heterogeneity of cerebral TDP-43 pathology in sporadic amyotrophic lateral sclerosis: Evidence for clinico-pathologic subtypes
Source: Acta Neuropathol Commun. 2016 Jun 23;4:61. doi: 10.1186/s40478-016-0335-2 (PMC4918136; doi:10.1186/s40478-016-0335-2)
Supplement: Additional file 3: Table S1. — Supplementary results on the neuropathological stages of Alzheimer’s disease-associated changes. (DOC 38 kb) [file 40478_2016_335_MOESM3_ESM.doc]

**Supplementary Table. Neuropathological stages of Alzheimer’s disease-associated changes**

|  | **Braak NFT stage**  [1, 2] | | | | | **Braak amyloid stage**  [2] | | | | **AD Neuropathologic change based on ‘ABC’ score** [1-5] | | | |
| --- | --- | --- | --- | --- | --- | --- | --- | --- | --- | --- | --- | --- | --- |
|  | 0 | I | II | III | IV-VI | 0 | A | B | C | Not | Low | Inter- mediate | High |
| **Type 1** | 13  (21%) | 20  (32%) | 23  (37%) | 7  (11%) | 0 | 34  (54%) | 13  (21%) | 10  (16%) | 6  (10%) | 34  (54%) | 28  (44%) | 1  (2%) | 0 |
| **Type 2a** | 3  (14%) | 7  (32%) | 10  (45%) | 2  (9%) | 0 | 10  (45%) | 3  (14%) | 6  (27%) | 3  (14%) | 10  (45%) | 11  (50%) | 1  (5%) | 0 |
| **Type 2b** | 1  (10%) | 4  (36%) | 5  (45%) | 1  (10%) | 0 | 4  (36%) | 4  (36%) | 1  (10%) | 2  (18%) | 4  (36%) | 6  (55%) | 1  (10%) | 0 |

ALS = amyotrophic lateral sclerosis, NFT = neurofibrillary tangle, AD = Alzheimer’s disease.

**Supplementary References**

1. Braak H, Alafuzoff I, Arzberger T, Kretzschmar H, Del Tredici K (2006) Staging of Alzheimer disease-associated neurofibrillary pathology using paraffin sections and immunocytochemistry. Acta Neuropathol 112:389-404
2. Braak H, Braak E (1991) [Neuropathological stageing of Alzheimer-related changes.](http://www.ncbi.nlm.nih.gov/pubmed/1759558) Acta Neuropathol 82:239-259
3. Mirra SS, Heyman A, McKeel D, Sumi SM, Crain BJ, Brownlee LM, Vogel FS, Hughes JP, van Belle G, Berg L (1991) The Consortium to Establish a Registry for Alzheimer's Disease (CERAD). Part II. Standardization of the neuropathologic assessment of Alzheimer's disease. Neurology 41:479-486
4. Montine TJ, Phelps CH, Beach TG, Bigio EH, Cairns NJ, Dickson DW, Duyckaerts C, Frosch MP, Masliah E, Mirra SS, Nelson PT, Schneider JA, Thal DR, Trojanowski JQ, Vinters HV, Hyman BT; National Institute on Aging; Alzheimer’s Association (2012) National Institute on Aging-Alzheimer's Association guidelines for the neuropathologic assessment of Alzheimer's disease: a practical approach. Acta Neuropathol 123:1-11. doi: 10.1007/s00401-011-0910-3
5. Thal DR, Rüb U, Orantes M, Braak H (2002) [Phases of A beta-deposition in the human brain and its relevance for the development of AD.](http://www.ncbi.nlm.nih.gov/pubmed/12084879) Neurology 58:1791-1800
